# Croup—Tracheotomy—Recovery

**Published:** 1861-03

**Authors:** Henry B. C. Greene


### *Croup—Tracheotomy—Recovery.*

On Friday, January 4th, I was called, at 10, A. M., to visit B——, aged 4½ years. On arrival, found her suffering from "membranous croup." The history of the case, as related by the parents, was as follows: "The Saturday night previous she appeared to have taken cold, and had a dry, hard cough. The next morning she appeared better, but commenced that night to be stuffed up and breathe with difficulty," which symptoms continued to increase until I saw her. She had had no medical attendance. She was then breathing with great difficulty; dry, sonorous inspiration; hoarse, and unable to speak above a whisper; lips blue; eyes suffused; pulse rapid and feeble; considerable jactitation, and tendency to drowsiness. I gave an emetic of sulph. cupri, which soon operated, but with no relief. I stated to the parents that in my opinion, the case was hopeless, as far as medical treatment was concerned, and suggested tracheotomy as affording the only, and that an extremely small chance for relief. At my request, they consented to have Dr. Kimball called in consultation. Her residence being several miles out of town, we were unable to get there until late in the afternoon, when we found the patient still more exhausted than in the morning, and concluded to operate at once. I opened the trachea with but little hæmorrhage; not having any tube expressly intended for the purpose, I substituted a piece of a large catheter, which answered very well. The patient did not seem at first to be any relieved, and we were not at all encouraged. We ordered tincture of veratrum viride, two drops every three hours; demulcent drinks; the atmosphere of the room to be kept moist; also gave directions to have the tube kept free with a feather, if it should become clogged.

Saturday, 5th, 10, A. M.—Found the patient slightly relieved; countenance better; pulse slower, and more full. During the morning, before I arrived, the tube had slipped out. I found the wound swollen, its edges everted and covered with a white exudation. The incision in the trachea was clear an open, air passing in readily. On taking everything into consideration, want of a proper instrument, &c., I concluded to let it alone so long as the wound in the trachea remained open and free. I gave another emetic of sulph. cupri, which in a few minutes expelled, by the mouth, several pieces of "false membrane," with about two ounces of very tenacious mucus. Ordered a cathartic of hyd. submur., ipecac. and rhei; to have the wound occasionally sponged with tepid water; continue the veratria every four hours.

Sunday, 6th, 2, P. M.—Not as well; breathes with more difficulty than yesterday, but not with so much effort as before operation. Countenance pale and anxious. No discoloration of lips, or inclination to sleep. Wound in trachea open; two or three small shred of "false membrane" protruding. Pulse more rapid and weak. Gave another emetic, which operated immediately, expelling some of the exudation with tenacious mucus. Appeared relieved after operation of the emetic. Ordered two grains carb. ammonia every hour; one teaspoonful of the following: ℞. Syr. ipecac., ℥j.; Syr. senegæ, syr. prun. Virg., aa ʒiv. M. To be repeated every two or three hours. Rubefacients to feet.

Monday, 7th.—Much better; slept quietly two hours during the night. Fever abated; skin moist; cough not so dry or urgent. Wound in trachea still open and free. Has some appetite; asked for, and may have, a cracker. Ordered olei ricini, half an ounce. The expectorant to be continued; also the sponging of the wound. Omit all other medicine. Directed great care to be used in order to keep the air in the room warm and moist.

Owing to sickness, I was unable to visit the patient again, but a messenger came daily to report her condition.

Tuesday, 8th.—Was quite bright and playful last evening. Slept well. Cough loose and expectorates freely; she is very hungry. Wound shows some disposition to close, but still admits air. Continue expectorant mixture p. r. n. May have light nutritious diet.

Wednesday, 9th.—Doing well. Cough loose and subsiding; spoke aloud to-day for the first time. The little patient continued rapidly to improve; the wound closed, and is now entirely well.

I cannot in justice close the report of this case without acknowledging the obligation I owe to Dr. Kimball, not only for his efficient aid in performing the operation, but for his prompt decision in regard to its expediency, without which the consent of the friends would with difficulty have been obtained. HENRY B. C. GREENE, M.D.

[*Boston Medical & Surgical Journal.*

